# American Medical Association

**Published:** 1873-04

**Authors:** 


AMERICAN MEDICAL ASSOCIATION.—As the time approaches for the meeting of the American Medical Association inquiries are being made as to route, fare, hotel accommodations, etc., etc. We are not sufficiently versed in railroad matters to give our readers any idea as to the best route by which to reach St. Louis, but on the important matter of fare we understand that no deductions will be made from the usual prices. Hotel accommodations can be had at reasonable rates.

